# Fast Detection of Striped Stem-Borer (*Chilo suppressalis* Walker) Infested Rice Seedling Based on Visible/Near-Infrared Hyperspectral Imaging System

**DOI:** 10.3390/s17112470

**Published:** 2017-10-27

**Authors:** Yangyang Fan, Tao Wang, Zhengjun Qiu, Jiyu Peng, Chu Zhang, Yong He

**Affiliations:** 1 College of Biosystems Engineering and Food Science, Zhejiang University, Hangzhou 310058, China; fanyangy@zju.edu.cn (Y.F.); wt0330@zju.edu.cn (T.W.); jypeng@zju.edu.cn (J.P.); yhe@zju.edu.cn (Y.H.); 2 Key Laboratory of Spectroscopy Sensing, Ministry of Agriculture, Hangzhou 310058, China

**Keywords:** rice, striped stem-borer, hyperspectral imaging, texture feature, data fusion

## Abstract

Striped stem-borer (SSB) infestation is one of the most serious sources of damage to rice growth. A rapid and non-destructive method of early SSB detection is essential for rice-growth protection. In this study, hyperspectral imaging combined with chemometrics was used to detect early SSB infestation in rice and identify the degree of infestation (DI). Visible/near-infrared hyperspectral images (in the spectral range of 380 nm to 1030 nm) were taken of the healthy rice plants and infested rice plants by SSB for 2, 4, 6, 8 and 10 days. A total of 17 characteristic wavelengths were selected from the spectral data extracted from the hyperspectral images by the successive projection algorithm (SPA). Principal component analysis (PCA) was applied to the hyperspectral images, and 16 textural features based on the gray-level co-occurrence matrix (GLCM) were extracted from the first two principal component (PC) images. A back-propagation neural network (BPNN) was used to establish infestation degree evaluation models based on full spectra, characteristic wavelengths, textural features and features fusion, respectively. BPNN models based on a fusion of characteristic wavelengths and textural features achieved the best performance, with classification accuracy of calibration and prediction sets over 95%. The accuracy of each infestation degree was satisfactory, and the accuracy of rice samples infested for 2 days was slightly low. In all, this study indicated the feasibility of hyperspectral imaging techniques to detect early SSB infestation and identify degrees of infestation.

## 1. Introduction

Rice is one of the most important foods for more than half of the global population. Pest infestation is one of the severe threats to rice growth, and it usually leads to serious loss of yield and quality [1]. Striped stem-borer (SSB) is one of the destructive rice pests in many rice-growing countries [2]. The traditional detection method for SSB is manual inspection according to conspicuous symptoms, such as a dead heart at tillering age and a white head at booting age [3]. As striped stem-borer is a boring insect and feeds on plant tissue in the stem wall [4], the stalk characteristics will change earlier than the canopy characteristics. Accurate SSB statistics need to dissect rice in the laboratory, which demands expert knowledge of the pest. This procedure is time-consuming and labor-intensive, and will decrease the detection efficiency and delay the appropriate controlling time. Hence, an efficient and effective detection method is necessary for early detection of SSB infestation in rice.

The optical properties of the plant refer to the absorption, reflectance and transmittance of light when the plant surface interacts with radiant energy. Reflectance can be influenced by the plant’s physiological properties and, thus, has been utilized by spectral technology to detect plant disease [5], fruit quality [6], agricultural product characteristics [7] and so on. Pest infestation can cause external and internal damage to the plant, such as the destruction of cell structure by the nibbling of tissue [8] and the loss of photosynthetic pigments by the piercing and sucking of sap [9], etc. The reflectance of plants in the visible waveband is associated with pigments, while reflectance in the near-infrared waveband provides information about plant water content and physical structure [10]. Thus, the spectral technique has the potential to be used to detect pest infestation in plants by measuring changes in spectral reflectance [11,12,13].

Besides changes in spectral characteristics, the external features of a plant would also change along with the changes of its physiological properties induced by pest feeding, excretion and other activities. These changes, such as yellowing, rot, defect, etc., could be captured by machine vision, and the image features would vary with the level of aggravation of the infestation. The extracted image features could be used to establish detection models with the reference data. Hence, imaging techniques have been applied in order to detect pest infestation [14,15,16].

Hyperspectral imaging is a technique integrating spectroscopy and imaging techniques, which can acquire both spectral and spatial information at high resolution. Hyperspectral imaging has been investigated as a potential technique in crop protection [17]. Sytar et al. [18] have reviewed the studies of hyperspectral imaging techniques to detect plant changes caused by salt stress and have shown the potential of this technique to detect salinity in soil. Thomas et al. [19] have reviewed research about plant disease detection based on the hyperspectral imaging technique and have shown the potential to detect and identify plant diseases before visible symptoms appear.

Zhao et al. [20] have distinguished Chinese cabbage infested by aphids from healthy cabbage based on hyperspectral imaging technology, and obtained the highest accuracy rate of 90%. Wu et al. [21] have employed the hyperspectral imaging technique to detect *Pieris rapae* larvae on cabbage leaves, and acquired classification accuracy above 96%. Thus, these studies indicated that the hyperspectral imaging technique has great potential for detecting pest infestation.

The main purpose of this study was to detect early striped stem-borer infestation in rice and identify degrees of infestation based on a visible/near-infrared hyperspectral imaging system. The specific objectives were to: (1) establish back-propagation neural network (BPNN) models to identify healthy rice samples and samples infested to different degrees; (2) select characteristic wavelengths by successive projection analysis (SPA); (3) extract textural features based on the gray-level co-occurrence matrix (GLCM); (4) improve detection performance by combining characteristic wavelengths and texture features.

## 2. Materials and Methods

### 2.1. Rice Samples Preparation

A total of 114 rice plants (Y Liangyou689, non-glutinous rice) were grown in an outdoor environment under insect-proof screen in Zhejiang University, Hangzhou, China. Eggs of the striped stem-borer were bought from the Shennong Biotechnology Company, Hangzhou, China, and were hatched on a moistened filter paper in a petri dish at a temperature of 28–30 °C and under illumination of 3000 lux for 10 h. When the rice was at the tillering stage, one first-instar striped stem-borer larva was placed on the rice after 2 h of starvation [22]; 69 rice plants were inoculated as the experimental group; and 45 rice plants were kept as the control group without inoculation.

**Figure 1:**
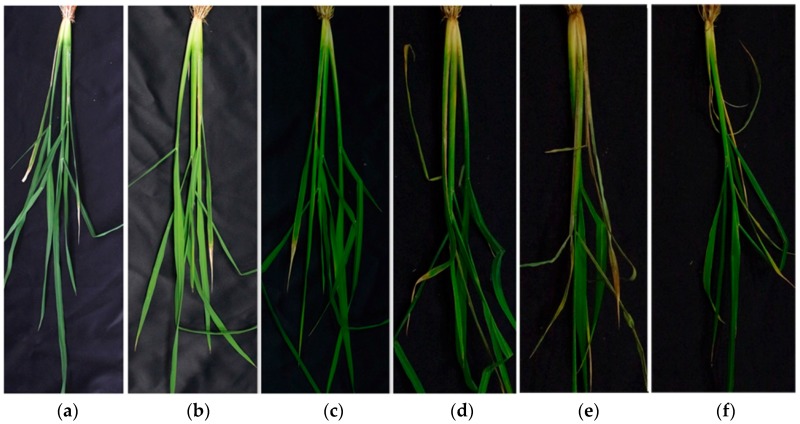
Samples of six degrees of infestation: (**a**) DI0; (**b**) DI1; (**c**) DI2; (**d**) DI3; (**e**) DI4; (**f**) DI5.

The hyperspectral images of control and infested rice plants were acquired every two days from 22 July 2016 (two days after infestation) to 30 July. The acquisition terminated on the eleventh day after infestation because the symptoms of top yellowing and stem-rotting lesions were already serious enough for there to be no need for detection by hyperspectral technology. The degrees of infestation (DI) were divided into DI1, DI2, DI3, DI4, DI5 according to the infested days, as shown in Figure 1; and the control group with healthy rice plants was referred as DI0. DI1 referred to the samples infested for two days, DI2 referred to the samples infested for 4 days, and so on. The number of infested samples on the last day decreased to 44 because of the loss caused by aggravated infestation. Thus, a total 365 samples (the number of DI0, DI1, DI 2, DI3, DI4, DI5 were 45, 69, 69, 69, 69, and 44 respectively) were acquired in this study.

### 2.2. Hyperspectral Imaging System and Image Acquisition

#### 2.2.1. Hyperspectral Imaging System

The visible/near-infrared hyperspectral imaging system, with 512 bands in the spectral ranges of 380–1030 nm, includes an imaging spectrograph (ImSpectorV10E; Spectral Imaging Ltd., Oulu, Finland); a 672 × 512 CCD camera (C8484-05, Hamamatsu Photonics, Hamamatsu, Japan); a camera lens (OLES23; Specim, Spectral Imaging Ltd., Oulu, Finland); two 150 W tungsten halogen lamps (Fiber-Lite DC950 Illuminator; Dolan Jenner Industries Inc., Boxborough, MA, USA) placed on both sides of the camera at a 45° angle; and a conveyer belt driven by a stepping motor (IRCP0076, Isuzu OpticsCrop, Zhubei, Taiwan). The system is controlled by a computer with Spectral Image-V10E software (Isuzu Optics Corp, Zhubei, Taiwan).

#### 2.2.2. Image Acquisition and Calibration

Before image acquisition, the parameters of the hyperspectral imaging system should be adjusted to acquire a clear and non-distorted image. The height between the samples and the lens was 35 cm; the conveyer belt’s moving speed was 3.00 mm/s; and the exposure time of the camera was 0.08 s. The sample was placed flat on the conveyer when collecting the image. To reduce noise and avoid the influence of dark current, the raw hyperspectral image should be calibrated according to the following formula:

(1) $$I_{c}=\frac{I_{\mathrm{raw}}-I_{\mathrm{dark}}}{I_{\mathrm{white}}-I_{\mathrm{dark}}}$$

where the $I_{c}$ is the calibrated hyperspectral image; $I_{\mathrm{raw}}$ is the raw hyperspectral image; $I_{\mathrm{dark}}$ is the dark reference image with 0% reflectance; and $I_{\mathrm{white}}$ is the white reference image with 99.9% reflectance.

### 2.3. Spectral Information Extraction

The region of interest (ROI) was predefined as the stalk region of rice in the image. As each pixel in the hyperspectral image corresponds to a spectral curve in full bands, the spectrum of all pixels in the ROI was averaged as representative of the sample. The samples were divided into a calibration set and a validation set by the Kennard–Stone (KS) algorithm [23] in a ratio of 2:1. There were 243 samples in the calibration set and 122 samples in the prediction set.

### 2.4. Texture Feature Extraction

Textural features have been utilized to reflect a plant’s physical characteristics such as firmness, color and roughness, which are related to the spatial arrangement of pixel intensity in an image. SSB infestation would influence not only the spectral characteristic but also the textural features of rice stalk. The gray-level co-occurrence matrix (GLCM) [24], as one of the most commonly used textural features in hyperspectral imaging, is defined as the relative frequency of occurrence of pixel pairs in a certain distance (*D*) and direction (*θ*) [25]. Eight descriptors of GLCM were chosen in this study, including mean, variance, homogeneity, contrast, dissimilarity, entropy, second moment, and correlation. Mean is the average grey level in the chosen image. Variance reflects the grey-level standard deviation. Homogeneity measures the closeness of the distribution of elements in the GLCM to the GLCM diagonal. Contrast is a measure of the degree of spread of the grey levels or the average grey-level difference between neighboring pixels. Dissimilarity is similar to contrast, but increases linearly as the difference between two pixels increases. Entropy measures the degree of disorder in an image. Second moment measures the textural uniformity or pixel-pair repetitions. Correlation is a measure of grey-level linear dependencies in the image [26,27].

Each band in a hyperspectral image corresponds to a gray-scale image, and one hyperspectral image contains 512 images. The textural features set will be huge and difficult to calculate if GLCM features are extracted based on full bands. Thus, principal component analysis (PCA) was employed to transform hundreds of images into principal component (PC) images, and the textural features were extracted based on the first few PC images that contained enough valid information. The extraction of spectral and textural features from hyperspectral images was performed on ENVI 4.6 (ITT, Visual Information Solutions, Boulder, CO, USA).

### 2.5. Data Analysis

#### 2.5.1. Characteristic Wavelength Selection

The hyperspectral data of each sample contains 512 variables in a full band. The redundancy and collinearity of the huge dataset would inevitably disturb the detection accuracy. A selection of characteristic wavelengths which have the most influence on the degree of infestation is essential in order to reduce the data dimensions and improve the detecting efficiency.

The successive projection algorithm (SPA) was employed in this study to choose the characteristic wavelengths. SPA is a common and effective method for reducing the variables of hyperspectral data, which can minimize the collinearity effects of raw input [28,29]. SPA is a forward variable selection method [30] by optimizing the multiple linear-regression (MLR) model, which includes two phases.

Phase 1 is to project the input *X* (*N* × *K*) matrix, and generate *K* chains with *M* variables, *M* = min(*N* − 1, *K*). This process includes six steps: Step 1 is to initialize z*^i^* with x*_k_*, where *i* is the iteration counter, and initialize $x_{j}^{i}$ with x*_j_*, where *j* = 1,…, *k*. Step 2 is to calculate the matrix *P* of projection onto the orthogonal subspace to z*^i^*. Step 3 is to calculate the projected vector $x_{j}^{i+1}$. Step 4 is to determine the index *j*_max_ of the largest projected vector and store the index. Step 5 is to initialize z*^i^*^+1^ with $x_{jmax}^{i+1}$. Step 6 is to return to Step 2 and start another iteration if *i* < M.

Phase 2 is to choose the best variable subset from the candidate subsets extracted from the *K* chains according to the minimum root mean square error (RMSE) obtained by applying the MLR model to the validation set [31].

#### 2.5.2. Chemometrics Algorithm

To identify degrees of infestation, the chemometrics algorithm needs to be employed as a classifier in order to accept the extracted information as input. The BPNN is a multi-layer feed-forward neural network with great capacity for non-linear mapping, and has been applied in hyperspectral imaging analysis in many studies [32,33,34]. The basic BPNN consists of an input layer, a hidden layer and an output layer. The connection between layers depends on the nodes of each layer. There are three main steps to train the BPNN model, including feed-forward computation, errors back-propagation, and weights updates [35]. Feed-forward computation aims to calculate and transmit the value of nodes in the order from the input layer to the output layer. Errors back-propagation aims to calculate the errors between the output and the reference and transmit the errors back successively. The weights are then updated until the error meets the target error or the training times reach the requirements. After comparing multiple network structures with different parameters, the optimal parameters of the number of nodes, the learning rate, the target error and the training times were set as 5, 0.6, 1 × 10^−5^, and 1000, respectively. Identification accuracy and run time were employed to evaluate the BPNN performance with different datasets. The SPA and BPNN algorithms were executed on Matlab R2011b (The Math Works, Natick, MA, USA).

## 3. Results

### 3.1. Spectra Features

**Figure 2:**
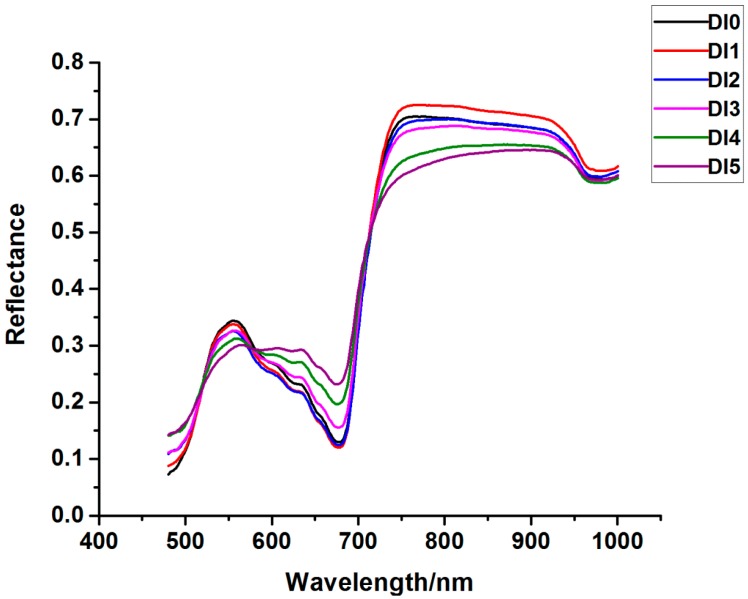
Average spectral curves of samples in six degrees of infestation.

The head and end ranges of wavebands contain a large proportion of noise. Therefore, the first 82 bands and the last 22 bands were removed to improve the signal-noise ratio. Spectra in the range of 480–1000 nm were pre-processed by Savitzky–Golay smoothing before analysis. The average spectra of each degree are shown in Figure 2. It was found that the general trends of six curves were similar. Significant differences of reflectance were observed in the range of 530–700 nm and 750–940 nm. The differences between the first three degrees were smaller than the differences between the last three degrees, which could be explained by the fact that damage symptoms of the samples in the first four days were mild and the stem structures were not destroyed too seriously. In the visible range of 570–700 nm, the reflectance was higher with the increase of infestation severity. This was because of the destruction of chlorophyll located in the chloroplast of the rice stem’s cortex cell [36]. In the near-infrared range of 750–1000 nm, the reflectance of DI0 was higher than that of the infested samples. The more severe the sample was infested, the lower the reflectance. The reduction of reflectance with the increase of severity was mainly due to the destruction of stem structure, which led to photon scattering [37].

As illustrated in Figure 2, the reflectance of DI1 and DI2 was lower than that of DI0 in the range of 570–700 nm, and the reflectance of DI1 and DI2 was higher than that of DI0 in the range of 750–1000 nm. This phenomenon may be explained by the compensation effect while pest infestation was in the incipient stage. In the early stage of striped stem-borer infestation, rice can generate a series of compensating responses to the injury, such as increasing the photosynthesis rate of healthy leaves, translocating photoassimilates from the damaged tillers to healthy tillers, and increasing productive tiller numbers [38,39]. But if the SSB cannot be controlled in a timely way, the compensating responses will not counteract the damage that SSB causes to rice, leading to serious yield loss.

### 3.2. Qualitative Analysis by PCA

PCA was employed in this study to investigate qualitatively the clustering trend of the samples based on full spectra. PCA can orthogonally transform the original possibly correlated variables into more uncorrelated variables that display the internal structure of the data [40]. The first principal component explained the largest variance, and the explained variables of the following PCs decreased successively. Thus, the first few PCs usually explain the most variances. In this study, PC1, PC2, and PC3 totally explained 98.3% of the variables, which were chosen to investigate the distribution pattern.

**Figure 3:**
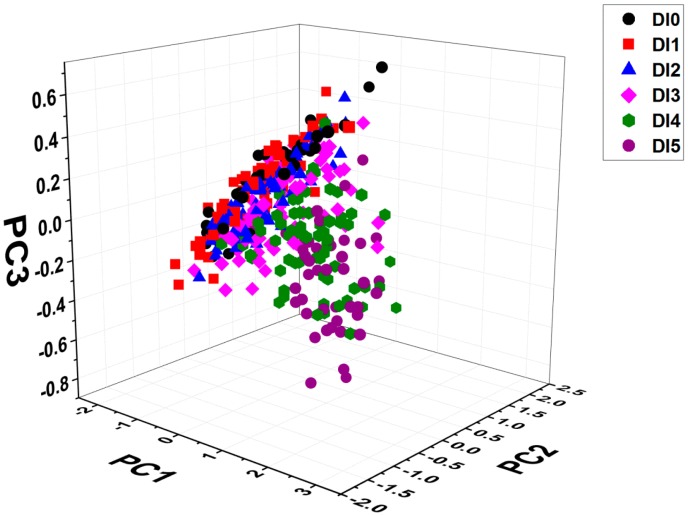
Scores’ scatter plots of samples in six degrees of infestation.

The three-dimensional scores’ scatter plot is displayed in Figure 3. In general, there was an obvious separation trend between the first four degrees and the last two degrees. The samples of the first four degrees were closely distributed while the samples of the last two degrees had a scattered distribution. The overlaps were serious between different degrees, but the samples of the first three degrees were seldom confused with the samples of the last two degrees. These phenomena indicated that the spectral characteristics would evidently change after DI3, and the chemometric method is necessary to identify accurately the degree of infestation.

### 3.3. Identification Results Based on Full Spectra

Full spectra were used as input to the BPNN model in order to identify different degrees of infestation. The results are given in Table 1. The overall accuracy was satisfactory, with classification accuracy over 90% for both the calibration and prediction sets. The classification accuracy of each degree was over 90% for both the calibration and prediction sets, except for DI0. The detection accuracy of DI1 was higher than DI0 but lower than DI2, DI3, DI4 and DI5. The main errors were attributed to the misclassification of the adjacent degree. A total of 9 samples and 6 samples were confused between DI0 and DI1 in the calibration and prediction sets, respectively.

It can be concluded from the results that the spectra combined with the BPNN algorithm was effective in identifying the degree of SSB infestation. Furthermore, early infestation was comparatively difficult to identify, which might because few changes of spectral characteristics occurred in this degree.

### 3.4. Characteristic Wavelengths Selection

**Figure 4:**
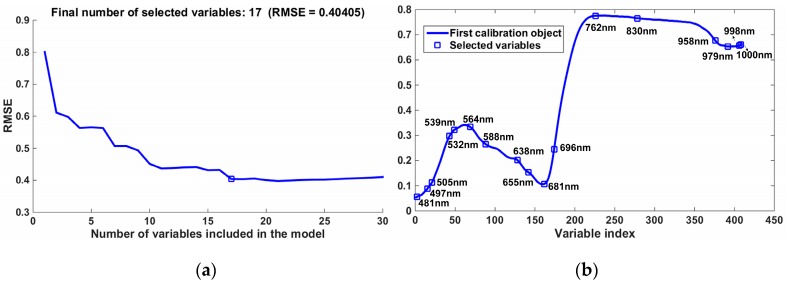
Selection of characteristic wavelengths by the successive projection algorithm (SPA): (**a**) numbers of characteristic wavelengths with the minimum root mean square error (RMSE); (**b**) distribution of characteristic wavelengths in the full band.

Hyperspectral images with hundreds of variables will result in information collinearity and redundancy, and slow the calculation efficiency. Thus, selecting the most informative variables will reduce the variables, obviously, and hence simplify the analysis. SPA was implemented in this study and selected a total of 17 variables (481, 497, 505, 532, 539, 564, 588, 638, 655, 681, 696, 762, 830, 958, 979, 998, and 1000 nm) from the entire 490 variables. The selected wavelengths are shown in Figure 4. The number of variables has decreased by more than 96%, which can simplify the detection models and improve the calculation efficiency.

As shown in Figure 4b, there were 11 wavelengths in the visible range and 6 wavelengths in the near-infrared range. The characteristic wavelengths in the visible range were mainly due to the alteration of photosynthetic pigments; for example, 532 nm was related to xanthophyll and 696 nm was largely sensitive to variation in chlorophyll content [41,42]. Furthermore, the characteristic wavelengths in the near-infrared range had a close relationship with the water content and internal structure of the rice [36]. These were correlated with destruction caused by the SSB, further indicating that characteristic wavelengths selected by SPA were meaningful in this study.

### 3.5. Identification Results Based on Characteristic Wavelengths

The results of the BPNN model based on characteristic wavelengths are shown in Table 2. On the whole, the overall accuracy of both calibration and prediction sets slightly decreased compared with the model based on full spectra. A better performance was achieved for detecting DI0. The results of each degree were promising, as accuracy was all beyond 90% except for DI1. The accuracy of DI0 and DI1 was lower than the rest of the degrees. Samples in DI2 and DI3, as well as in DI4 and DI5, were more easily confused with each other. However, misclassification between DI3 and DI4 occurred rarely, which was consistent with the clustering trend in the PCA analysis. These results indicated that the selection of characteristic wavelengths by SPA was effective at both maintaining performance and reducing variables. The spectral characteristics of the samples in adjacent degrees was similar, and this would increase the difficulty in identifying the degree of infestation accurately.

### 3.6. Identification Results Based on Textural Features

**Figure 5:**
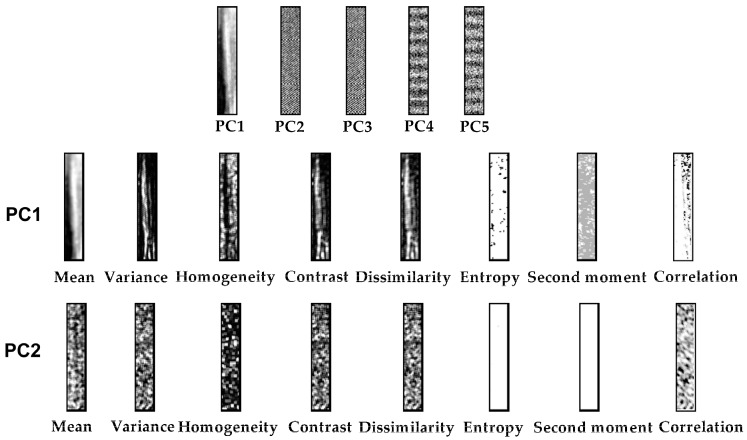
Texture feature images and PC images of a rice sample.

The hyperspectral image contains 512 gray-scale images according to the dimension of wavelengths, and it would be better to compress these into fewer images. Therefore, PCA was performed on the ROI hyperspectral image to extract the most informative PC images. The PC1 and PC2 images comprised a total 93.42% of the eigenvalues, and the rest of the PC images contained more noise than information, which would disturb the detection. Thus, only the PC1 and PC2 images were retained to extract the GLCM features. There were a total of 8 features for each image including mean, variance, homogeneity, contrast, dissimilarity, entropy, second moment, and correlation; thus, a new features set was formed for 16 features of each sample, as shown in Figure 5. Multiple linear-regression analysis was executed to inspect the relationship between textural features and infestation degrees. The multiple correlation coefficient *R* was 0.811 and the coefficient of determination *R*^2^ was 0.658, which meant the prediction was satisfactory. These demonstrated that the GLCM features contained useful information, which could be helpful for identification of SSB infestation.

The new dataset was used as input for the BPNN, and the results are shown in Table 3. The identification accuracy of the DI0 and DI4 and DI5 was all over 80% in the prediction set, while the accuracy of the DI1-DI3 was relatively low. Furthermore, the rice samples in medium infestation degrees were similar with respect to the textural features, and hence had a greater possibility of being confused with each other. However, the overall results proved that the GLCM features were worthy of further exploration combined with spectral data.

### 3.7. Identification Results Based on Data Fusion

The fusion of spectral data and textural features has been explored by many studies into food quality and plant-disease detection based on hyperspectral imaging technology [43,44,45]. Data fusion can be performed at three levels: pixel-level fusion, feature-level fusion, and decision-level fusion [46]. Data fusion at the feature level means extracting a feature from different data sets and fuses statistical approaches such as arithmetic combinations and filters [47]. This study adopted feature-level fusion to fuse the characteristic wavelengths with textural features. Spectral and textural features were also normalized in the same dimension before fusion.

The results of the BPNN model using data fusion are shown in Table 4. The identification results of both calibration and prediction sets were excellent, with classification accuracy over 95%. Meanwhile, the performance in detecting each infestation degree was satisfactory, with classification accuracy over 95%, except for DI1. The cause of this error was misclassification with DI0, which was consistent with the phenomenon discussed in the above sections.

### 3.8. Comparision of BPNN Models Based on Different Datesets

In this study there were four data sets—including full spectra, characteristic wavelengths, textural features and data fusion—as input for the BPNN model to detect different degrees of SSB infestation. The total accuracy of the BPNN models based on full spectra and characteristic wavelengths was higher than models based on textural features. This indicated that the spectral features contributed more to the identification of infestation than the textural features. The accuracy of the BPNN models based on characteristic wavelengths decreased in comparison with the BPNN models based on full spectra, which might be connected with the loss of certain useful information after extracting 17 wavebands from the full 408 wavebands. The BPNN models based on data fusion acquired the highest total accuracy among the four data sets, which could not only remedy the deficiency of the set of individual characteristic wavelengths or textural features, but also improve the calculation efficiency, as the run time decreased by about 90% compared with models based on the full spectra set in Table 5. The accuracy of individual degrees was all elevated by the fusion of characteristic wavelengths and textural feature; even the accuracy of DI1 exceeded 80%. These results indicate that the spectral and textural features were complementary in internal and external aspects, which could reflect integrated changes of plant characteristics. The fusion of spectral and textural features could take full advantage of hyperspectral imaging technology, and was effective at detecting SSB infestation and identifying different degrees of it.

## 4. Conclusions

This study explored the feasibility of using a visible/near-infrared hyperspectral imaging system to detect early SSB infestation and identify degrees of infestation in rice. We selected 17 characteristic wavelengths by SPA from full spectral data, and extracted 8 GLCM features from the PC images transformed from hyperspectral images. BPNN models were established using different data sets, including full spectra, characteristic wavelengths, textural features and features fusion. The BPNN model based on feature fusion acquired the best results, with overall accuracy over 95%. The identification accuracy of each infestation degree was over 95%, except for DI1, which was also improved compared to models using characteristic wavelengths and textural features alone. The run time decreased significantly as a result of variables selection. DI1 was easily confused with DI0, which increased the difficulty of the early detection of SSB infestation. In total, these results proved that the fusion of spectral and textural features from hyperspectral images combined with BPNN was feasible for identifying degrees of SSB infestation in rice. In future studies, we will develop more stable and universal models with more rice cultivars and more species of pests for laboratory-based detection. Field-based research will also be conducted on the basis of this laboratory-based research in order to expand the application of hyperspectral imaging combined with chemometrics in the field. Small and portable pest-detection equipment will be developed based on future work.

## Figures and Tables

**Table 1:** Detection accuracy of six infestation degrees by the BPNN model based on full spectra.

Model	Actual Value	Calibration Set							Prediction Set						
		DI ^1^0	DI1	DI2	DI3	DI4	DI5	Accuracy	DI0	DI1	DI2	DI3	DI4	DI5	Accuracy
BPNN	DI0	23	6	0	0	1	0	76.67%	9	5	0	0	1	0	60%
	DI1	3	43	0	0	0	0	93.48%	1	22	0	0	0	0	95.65%
	DI2	0	0	46	0	0	0	100%	0	0	23	0	0	0	100%
	DI3	1	0	0	44	0	1	95.65%	0	0	0	22	0	1	95.65%
	DI4	0	0	0	0	46	0	100%	0	0	0	0	23	0	100%
	DI5	0	0	0	0	0	29	100%	0	0	0	0	0	15	100%
	Total							95.06%							93.44%

^1^ Degree of infestation.

**Table 2:** Detection accuracy of infestation degrees by the BPNN model based on characteristic wavelengths.

Model	Actual Value	Calibration Set							Prediction Set						
		DI ^1^0	DI1	DI2	DI3	DI4	DI5	Accuracy	DI0	DI1	DI2	DI3	DI4	DI5	Accuracy
BPNN	DI0	24	2	1	0	3	0	80%	13	0	1	0	1	0	86.67%
	DI1	5	40	0	0	1	0	86.96%	4	18	0	0	1	0	78.26%
	DI2	0	0	45	1	0	0	97.83%	0	0	22	1	0	0	95.65%
	DI3	0	0	1	44	0	1	95.65%	0	0	0	22	0	1	95.65%
	DI4	0	1	0	0	44	1	95.65%	0	0	0	0	22	1	95.65%
	DI5	0	0	0	0	1	28	96.55%	0	0	0	0	1	14	93.33%
	Total							92.59%							90.98%

^1^ Degree of infestation.

**Table 3:** Detection accuracy of infestation degrees by the BPNN model based on the GLCM features.

Model	Actual Value	Calibration Set							Prediction Set						
		DI ^1^0	DI1	DI2	DI3	DI4	DI5	Accuracy	DI0	DI1	DI2	DI3	DI4	DI5	Accuracy
BPNN	DI0	30	0	0	0	0	0	100%	15	0	0	0	0	0	100%
	DI1	9	25	3	5	3	1	54.35%	3	12	2	3	3	0	52.17%
	DI2	0	1	32	10	1	2	69.57%	0	0	16	4	1	2	69.57%
	DI3	0	5	10	26	2	3	56.52%	0	2	6	14	0	1	60.87%
	DI4	0	0	2	1	38	5	82.61%	0	0	1	0	19	3	82.61%
	DI5	0	0	0	4	2	23	79.31%	0	0	0	2	0	13	86.67%
	Total							71.60%							72.95%

^1^ Degree of infestation.

**Table 4:** Detection accuracy of infestation degrees by the BPNN model based on data fusion.

Model	Actual Value	Calibration Set							Prediction Set						
		DI ^1^0	DI1	DI2	DI3	DI4	DI5	Accuracy	DI0	DI1	DI2	DI3	DI4	DI5	Accuracy
BPNN	DI0	29	1	0	0	0	0	100%	15	0	0	0	0	0	100%
	DI1	4	42	0	0	0	0	82.61%	4	19	0	0	0	0	82.61%
	DI2	0	1	45	0	0	0	95.65%	0	1	22	0	0	0	95.65%
	DI3	0	0	1	44	1	0	100%	0	0	0	23	0	0	100%
	DI4	0	1	0	2	43	0	95.65%	0	0	0	1	22	0	95.65%
	DI5	0	0	0	0	1	28	100%	0	0	0	0	0	15	100%
	Total							95.06%							95.10%

^1^ Degree of infestation.

**Table 5:** Run time of the BPNN models based on different data sets.

Data Set	Run Time
Full spectra	16.91s
Characteristic wavelength	3.86 s
Texture features	1.64 s
Data fusion	1.80 s
